# Knowledge, awareness, and attitudes toward oral irrigator use among dentists and dental students: a cross-sectional study

**DOI:** 10.1186/s12903-026-07956-w

**Published:** 2026-02-26

**Authors:** Sema Merve Altıngöz, Sinem Coşkun Albayrak

**Affiliations:** 1 Department of Periodontology, Faculty of Dentistry, Lokman Hekim University, Cankaya, Ankara, 06530 Turkey; 2 Department of Oral and Maxillofacial Radiology, Faculty of Dentistry, Lokman Hekim University, Ankara, Turkey

**Keywords:** Oral irrigators, Oral hygiene, Interdental cleaning, Dental education, Preventive dentistry, Public health

## Abstract

**Background:**

Interdental plaque control is critically important in maintaining oral hygiene. Oral irrigators (also known as water flossers) are recommended as an alternative oral hygiene tool, especially for individuals who have difficulty using traditional dental floss. Although numerous studies evaluate the clinical effectiveness of oral irrigators in the literature, studies examining the knowledge level, awareness, and clinical attitudes of dentists and dental students towards these devices are limited. The aim of this study is to evaluate knowledge, awareness, and attitudes regarding the use of oral irrigators and to examine the effect of education and clinical experience levels on these approaches.

**Methods:**

A total of 421 participants were included in the study: third-year dental students (*n* = 100), intern dentists (*n* = 179), general practitioners (*n* = 66), and specialist dentists (*n* = 75). A structured questionnaire consisting of 16 questions was administered to assess knowledge, awareness, and attitudes regarding oral irrigator use. Data were analyzed using chi-square tests and Bonferroni-corrected post-hoc analyses; the statistical significance level was set at *p* < 0.05.

**Results:**

82.9% of participants reported having knowledge about oral irrigators. It was found that specialist and general practitioners had significantly higher knowledge levels regarding the periodontal effects of oral irrigators compared to students and interns (*p* < 0.001). 70.2% of participants rated oral irrigators as “effective” or “very effective”. While the rate of regular use was 9.0%, cost (58.0%**)** was identified as the primary barrier, whereas difficulty of use was reported by only a small proportion of participants (4.8%) (*p* < 0.001). Only 28.9% of participants stated that they had received adequate training about oral irrigators. The rate of specialist dentists recommending oral irrigators to their patients was significantly higher (61%) compared to other groups (*p* < 0.001).

**Conclusion:**

This study reveals that knowledge, awareness, and attitudes towards oral irrigator use differ significantly depending on the level of education and clinical experience. While general awareness is high, significant gaps exist in terms of detailed knowledge, personal use, and curriculum adequacy. To effectively integrate oral irrigators into preventive dentistry practices, it is recommended that evidence-based and structured training on these devices be strengthened in dental education programs.

**Supplementary Information:**

The online version contains supplementary material available at 10.1186/s12903-026-07956-w.

## Background

Dental plaque is an organized biofilm composed of various microorganisms that have strong adhesion to tooth surface and to each another [1]. The accumulation of dental plaque plays a key etiologic role in the development of dental caries and periodontal diseases, which remain among the most prevalent oral health problems worldwide [2]. Thus, maintaining oral health requires daily dental plaque control [3].

Devices that help the patient remove biofilm from all areas surrounding the teeth should be part of a patient’s daily oral hygiene routine [4, 5]. Toothbrushing is considered the most common and reliable method for mechanical plaque removal. Its effectiveness is largely limited; only supragingival plaque on lingual and facial surfaces can be removed by brushing [6]. Because it is difficult for the bristles to penetrate interdental gaps, the toothbrush cannot remove subgingival plaque in areas between teeth [7]. Most of the periodontal problems and caries begin in interdental areas as these spaces are difficult to clean. Therefore, removing the plaque from interproximal areas is crucial [8].

The best way to remove interdental plaque is with interdental cleaning aids. The selection of interdental aids is influenced by the type of embrasure and the motivation to use such aids [9]. Dental floss, interdental brushes, plastic dental picks, wooden interdental aids, and oral irrigators, also known as water flossers and dental water jets are interdental aids that are commonly used [10]. The “gold standard” for eliminating interdental plaque is dental floss [11]. However, using dental floss correctly takes time, demands experience, and is technique-sensitive [12, 13]. In order to facilitate interdental cleaning, novel products like water flossers-also referred to as water jets or oral irrigators-have been developed [6]. The effectiveness of oral irrigators has been compared in research to that of other interdental cleaning aids, including dental floss, and interdental brushes [14–21]. It was reported that water flossers are superior to regular flossing [12–14]. Furthermore, oral irrigators are easier to use and more pleasant than traditional flossing, particularly for those who have dental restorations or orthodontic treatment [15, 17, 22–24]. Previous studies have shown that oral irrigators are safe; there is no evidence of negative effects on the attachment or junctional epithelium [25]. Clinical studies have demonstrated that oral irrigators are effective and well tolerated, particularly among individuals with orthodontic appliances, dental implants, or limited manual dexterity. These findings have been supported by both randomized clinical trials and recent systematic reviews [10, 19].

The dual mechanisms of an oral irrigator are pulsation and water pressure. This power-driven device emits a controlled pulsating stream of water (typically within a pressure range of 50–90 psi) that effectively eliminates subgingival and interdental plaque. The synergistic action of pulsation and pressure helps disrupt bacterial biofilms and flush away loosely attached debris from the tooth surfaces, while remaining safe for the surrounding soft tissues [26].

Oral irrigators have become increasingly popular as an adjunctive oral hygiene tool, particularly for individuals with specific dental needs and for those seeking alternatives to traditional flossing. When used alongside regular toothbrushing and professional dental care, oral irrigators can contribute to effective plaque removal. Patient education on appropriate use and integration of oral irrigation into daily oral hygiene routines may therefore support the maintenance of oral health [26].

Although numerous studies in the literature examine the clinical effects of water jet (oral irrigator) devices on plaque control and gingival inflammation, studies evaluating the knowledge, awareness, and clinical attitudes of dentists and dental students towards these devices are limited. In particular, how perceptions and practices regarding water jet use differ among individuals with varying levels of education and clinical experience has not been sufficiently explored. This lack of information makes it difficult to standardize evidence-based oral hygiene recommendations in clinical practice.

The aim of this study was to evaluate the knowledge, awareness, and attitudes toward oral irrigator use and its contribution to oral hygiene among dentists and dental students, and to explore how educational background influences professional perspectives on this adjunctive oral hygiene method.

## Methods

### Study population

This descriptive cross-sectional study was conducted at Lokman Hekim University, Faculty of Dentistry. Ethical approval for the study was obtained from the Lokman Hekim University Ethics Committee (No: 2025/225) The minimum sample size for this descriptive cross-sectional survey was estimated using the single-proportion formula: *n* = Z² × *p*(1 − *p*) / *d*². Assuming a 95% confidence level (Z = 1.96), a conservative expected proportion of 50% (*p* = 0.50) to maximize sample size, and a margin of error of 5% (*d* = 0.05), the required minimum sample size was calculated as 384 participants. To account for potential non-response/incomplete questionnaires, the target sample was increased by approximately 10% (≈ 423).

A total of 421 participants were recruited to the study. The sample size included all the participants who met the inclusion criteria. Inclusion criteria were based on dentists and dental students who volunteered to participate in the study. Dental students were from third-, fourth- and fifth-year dentistry students. The study population comprised four professional subgroups:

Dental students (third-year) who had completed theoretical courses but not yet entered clinical training (*n* = 100), intern dentists (fourth- and fifth-year) actively treating patients under supervision (*n* = 179), general dentists (*n* = 66) and specialist dentists (*n* = 75).

### Questionnaire design

A structured questionnaire consisting of 16 items (15 single-choice and 1 multiple-choice question) was administered to the participants. The questionnaire was administered in Turkish, reflecting the native language of the target population. The questionnaire was specifically developed for this study based on a review of the existing literature on oral irrigators and adjunctive oral hygiene devices [27, 28]. Relevant domains were identified from previously published surveys and clinical studies, and an original set of items was constructed in line with the aims of the present study. The questionnaire was not directly adapted from a single validated instrument. Instead, content validity was established through alignment with previously published literature and expert review (Supplementary File 1). Items were generated to reflect key thematic domains identified in the literature and were evaluated by experts for relevance and clarity.

Face validity was assessed during pilot administration to ensure comprehensibility and feasibility of the questionnaire in the target population. As the instrument included heterogeneous domains and response formats (knowledge and behavior items, multiple-choice questions, and attitudinal statements), internal consistency reliability analysis was performed only for the attitudinal items measured using an agree/unsure/disagree response format, which represented a single conceptual construct.

A pilot study was conducted with 30 participants (10 dental students, 10 general dentists, and 10 specialist dentists) to evaluate the internal consistency of the attitudinal domain prior to final data collection. All questions were concise and easy to understand. The final form was distributed online via Google Forms, and invitations were sent through e-mail and WhatsApp channels to ensure wide participation. No personally identifiable information or IP addresses were collected, and responses were recorded anonymously and analyzed in aggregate. Access to the survey data was restricted to the research team via a password-protected account. An information and consent page was provided at the beginning of the survey, and e-survey reporting was guided by the CHERRIES checklist. Participants were recruited using a snowball sampling approach. The survey link was initially distributed to dental students at our university, as well as to general dentists and specialist dentists through professional and academic communication networks. All recipients were encouraged to forward the link to other eligible peers within their networks.

At the beginning of the questionnaire, an information page explained the study purpose, voluntary nature of participation, confidentiality, and the right to withdraw at any time without providing a reason. Participants provided informed consent electronically by selecting the ‘I agree to participate’ option before proceeding to the survey items. Participants could withdraw at any point by exiting the survey; no penalties were incurred, and incomplete questionnaires were excluded from analysis. For student participants, participation or withdrawal had no impact on course grades, examinations, or academic evaluation. Participants were asked to respond only once, and duplicate submissions were automatically filtered by Google Forms.

 The questionnaire was designed to evaluate three primary domains:

- Knowledge — understanding of the mechanism and purpose of oral irrigator use,
- Awareness — perceptions of its role in oral and periodontal health, and
- Attitude — personal experience, recommendation behavior, and educational perspectives.

### Statistical analyses

Statistical analysis of the data was carried out via the IBM Statistics SPSS 25 package program. All statistical analyses were conducted using chi-square tests to assess associations between categorical variables. Post-hoc comparisons with Bonferroni corrections were applied to identify group differences when overall significance was detected. Statistical significance was set at *p* < 0.05.

Descriptive statistics were used to summarize the distribution of responses across the four groups — dentistry students, intern dentists, general dentists, and specialist dentists. Chi-square analyses were conducted to evaluate relationships between professional groups and their familiarity with oral irrigators, knowledge of their periodontal effects, perceived effectiveness, personal and clinical usage patterns, and attitudes toward patient recommendation.

## Results

This questionnaire-based study provides comprehensive data on dentists’ and dental students’ knowledge, awareness, and attitudes toward oral irrigator use across different stages of dental education and clinical practice. A total of 421 participants completed the survey and were included in the analysis. The study population comprised 100 dentistry students (3rd year), 179 intern dentists (4th–5th year), 66 general dentists, and 75 specialist dentists. In order to identify significant differences between the groups, chi-square analyses and Bonferroni-corrected post-hoc comparisons were performed. In the pilot study (*n* = 30), the attitudinal items demonstrated good internal consistency (Cronbach’s α = 0.79). In the final study sample (*n* = 421), Cronbach’s alpha for the same attitudinal domain was 0.64, indicating acceptable internal consistency for the full dataset.

The results are presented according to the main thematic domains of the questionnaire.

The findings demonstrate clear variations among professional groups in terms of familiarity with oral irrigator use, understanding of its mechanism of action, perceived periodontal benefits, personal use, and attitudes toward recommendation in clinical practice. Several domains, including familiarity level, knowledge of periodontal effects, perceived effectiveness, barriers to use, and recommendation behavior, showed statistically significant intergroup differences, highlighting the influence of educational level and clinical experience on knowledge, awareness, and attitudes toward oral irrigators. (Table 1)

**Table 1:** Distribution of survey responses across professional groups

Survey question	Answer option	Dental Student *n* = 100	Intern Dentist *n* = 179	General Dentist *n* = 66	Specialist Dentist *n* = 76	*p* value
Do you have knowledge about oral irrigators (water flossers)?	Yes	59 (59.0)	159 (88.8)	64 (97.0)	68 (89.5)	**< 0.001**
	No	15 (15.0)	10 (5.6)	1 (1.5)	2 (2.6)	
	I am not sure	26 (26.0)	10 (5.6)	1 (1.5)	6 (7.9)	
How would you describe the primary function of an oral irrigator (water flosser)?	An alternative to toothbrushing	6 (6.0)	6 (3.4)	1 (1.5)	0 (0.0)	**< 0.001**
	An adjunctive method that supports toothbrushing	81 (81.0)	166 (92.7)	62 (93.9)	76 (100.0)	
	A device used only for orthodontic patients	0 (0.0)	1 (0.6)	1 (1.5)	0 (0.0)	
	I have no idea	13 (13.0)	6 (3.4)	2 (3.0)	0 (0.0)	
Do you have knowledge regarding the effects of oral irrigators (water flossers) on periodontal health?	Yes, I have detailed knowledge	4 (4.0)	31 (17.3)	26 (39.4)	30 (39.5)	**< 0.001**
	Yes, I have partial knowledge	63 (63.0)	133 (74.3)	40 (60.6)	43 (56.6)	
	No	33 (33.0)	15 (8.4)	0 (0.0)	3 (3.9)	
In your opinion, for which patient groups are oral irrigators particularly beneficial? (You may select more than one option)	Individuals undergoing orthodontic treatment	48 (48.0)	121 (67.6)	53 (80.3)	64 (84.2)	This was a multiple-response item; therefore, no overall p value was calculated.
	Dental implant patients	25 (25.0)	106 (59.2)	51 (77.3)	61 (80.3)	
	Individuals with periodontal problems	50 (50.0)	133 (74.3)	50 (75.8)	61 (80.3)	
	Children	20 (20.0)	63 (35.2)	20 (30.3)	15 (19.7)	
	Individuals with physical and/or intellectual disabilities	19 (19.0)	88 (49.2)	37 (56.1)	47 (61.8)	
	General population (everyone)	63 (63.0)	119 (66.5)	43 (65.2)	52 (68.4)	
	Other	10 (10.0)	10 (5.6)	5 (7.6)	1 (1.3)	
To what extent do you consider oral irrigators to be an effective oral hygiene method?	Very effective	16 (16.0)	31 (17.4)	13 (19.7)	17 (22.4)	**0.015**
	Effective	50 (50.0)	103 (57.9)	44 (66.7)	47 (61.8)	
	Partially effective	22 (22.0)	35 (19.7)	7 (10.6)	11 (14.5)	
	Ineffective	0 (0.0)	3 (1.7)	1 (1.5)	0 (0.0)	
	I have no idea	12 (12.0)	6 (3.4)	1 (1.5)	1 (1.3)	
Do you think the routine use of oral irrigators should be recommended in dental practice?	Yes	69 (69.0)	132 (73.7)	47 (71.2)	49 (64.5)	0.123
	No	1 (1.0)	12 (6.7)	5 (7.6)	7 (9.2)	
	I am undecided	30 (30.0)	35(19.6)	14(21.2)	20 (26.3)	
Have you ever used an oral irrigator?	Yes, I use it regularly.	5 (5.0)	16 (8.9)	11 (16.7)	6 (7.9)	0.107
	Yes, I use it occasionally.	18 (18.0)	37 (20.7)	16 (24.2)	21 (27.6)	
	No, I have never used it.	77 (77.0)	126 (70.4)	39 (59.1)	49 (64.5)	
If you have used an oral irrigator, for what purpose did you use it?	During orthodontic treatment	2 (2.0)	6 (3.4)	1 (1.5)	0 (0.0)	0.187
	For peri-implant care	1 (1.0)	2 (1.1)	1 (1.5)	0 (0.0)	
	For controlling gingival bleeding/plaque	1 (1.0)	4 (2.2)	4 (6.1)	1 (1.3)	
	For general oral hygiene	25 (25.0)	43 (24.0)	24 (36.4)	28 (36.8)	
	I have not used it	71 (71.0)	124 (69.3)	36 (54.5)	47 (61.8)	
After using an oral irrigator, my mouth feels cleaner and fresher.	I agree	23 (23.0)	55 (37.4)	26 (39.4)	27 (36.0)	0.017
	I disagree	2 (2.0)	1 (0.6)	0 (0.0)	0 (0.0)	
	I am not sure	5 (5.0)	1 (3.4)	4 (6.1)	0 (0.0)	
	I have not used it	70 (70.0)	122 (58.7)	36 (54.5)	48 (64.0)	
Oral irrigators are effective in reducing halitosis (bad breath).	I agree	66 (66.0)	134 (74.9)	49 (74.2)	59 (77.6)	0.434
	I disagree	2 (2.0)	5 (2.8)	3 (4.5)	1 (1.3)	
	I am not sure	32 (32.0)	40 (22.3)	14 (21.2)	16 (21.1)	
Do you recommend oral irrigators to your patients?	Yes, frequently	19 (19.2)	32 (18.0)	22 (33.3)	28 (36.8)	**< 0.001**
	Yes, in specific situations	49 (49.5)	112 (62.9)	39 (59.1)	34 (44.7)	
	No	31 (31.3)	34 (19.1)	5 (7.6)	14 (18.4)	
How do you think oral irrigator use affects patient compliance?	Positively	58 (58.0)	121 (68.4)	41 (62.1)	47 (62.7)	**0.001**
	Neutral	10 (10.0)	26 (14.7)	20 (30.3)	9 (12.0)	
	Negatively	1 (1.0)	2 (1.1)	0 (0.0)	1 (1.3)	
	I have no idea	31 (31.0)	28 (15.8)	5 (7.6)	18 (24.0)	
What do you think is the most significant factor limiting the use of oral irrigators?	Cost	40 (40.0)	111 (62.0)	43 (65.2)	50 (65.8)	**< 0.001**
	Device availability	10 (10.0)	15 (8.4)	5 (7.6)	4 (5.3)	
	Difficulty of use	2 (2.0)	10 (5.6)	5 (7.6)	3 (3.9)	
	Time-consuming	11 (11.0)	16 (8.9)	7 (10.6)	7 (9.2)	
	Lack of belief in its effectiveness	9 (9.0)	14 (7.8)	5 (7.6)	5 (6.6)	
	I have no idea	28 (28.0)	13 (7.3)	1 (1.5)	7 (9.2)	
Do you think you received sufficient education on oral irrigators during your undergraduate dental training?	Yes	13 (13.0)	40 (22.5)	13 (19.7)	3 (3.9)	**< 0.001**
	No	55 (55.0)	83 (46.6)	37 (56.1)	66 (86.8)	
	Partially	32 (32.0)	55 (30.9)	16 (24.2)	7 (9.2)	
I believe that the use of oral irrigators increases patients’ motivation for maintaining oral hygiene.	I agree	65 (65.0)	143 (81.2)	51 (77.3)	55 (72.4)	0.052
	I disagree	2 (2.0)	5 (2.8)	3 (4.5)	2 (2.6)	
	I am not sure	33 (33.0)	28 (15.9)	12 (18.2)	19 (25.0)	

### Knowledge

**Fig. 1:**
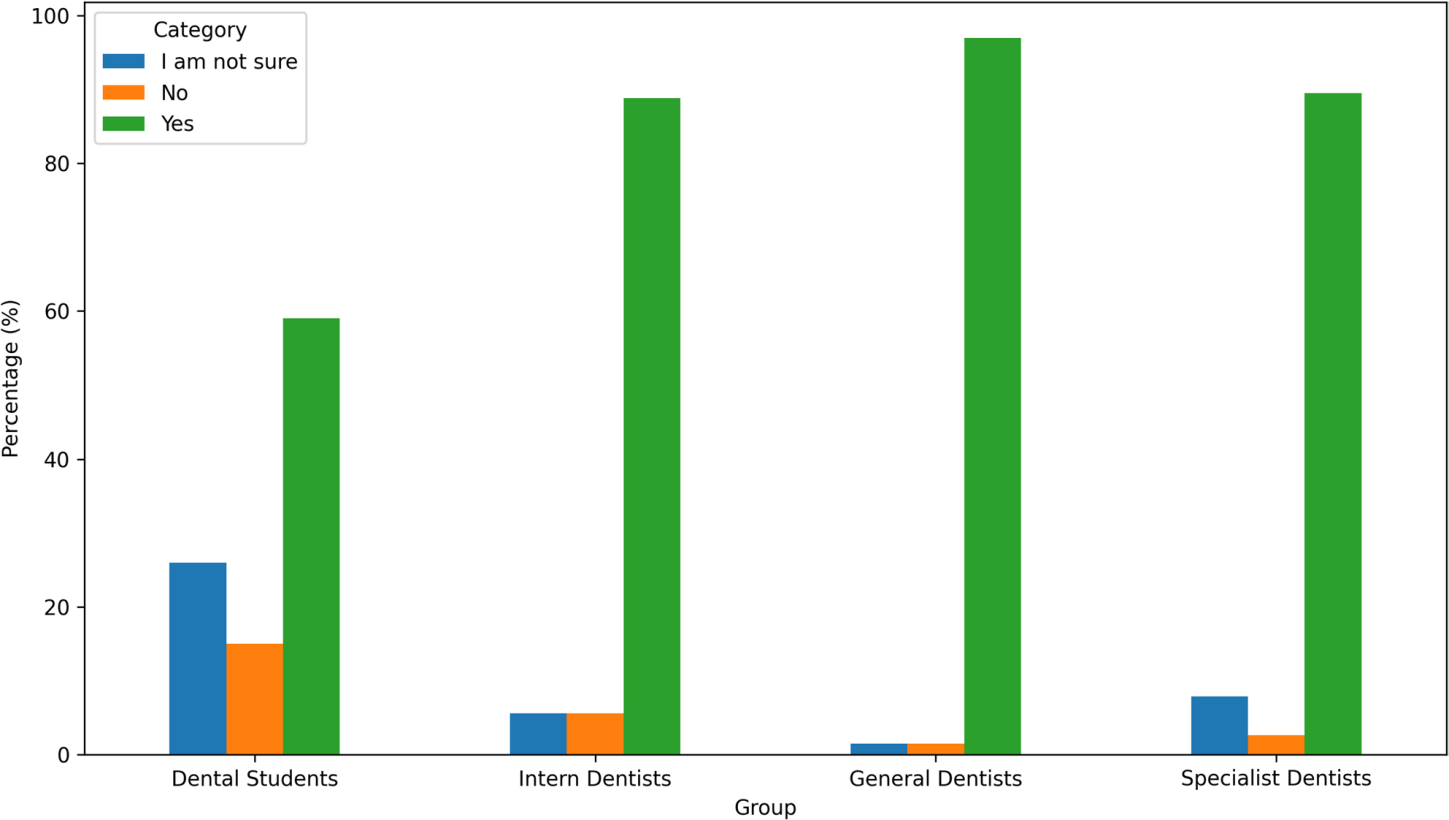
Familiarity with oral irrigator use by group

Overall, 83.1%, *n* = 350 of participants reported being familiar with oral irrigators. Significant differences were found among professional groups (*p* < 0.001). Specialist dentists and general dentists demonstrated higher familiarity levels compared to intern dentists and students. (Fig. 1)

A significant association was found between professional group and knowledge regarding the periodontal benefits of oral irrigator use (*p* < 0.001). While 96.1%, *n* = 73 of specialist dentists and 100%, *n* = 66 of general dentists reported partial or detailed knowledge of the device’s effects on periodontal health, only 91.6%, *n* = 164 of interns and 67.0%, *n* = 67 of students did so (Table 2).

**Table 2:** Summary of knowledge, awareness, and attitudes toward oral irrigator usage

Adequate undergraduate education (Yes)	Dental Student *n* (%)	Intern Dentist *n* (%)	General Dentist *n* (%)	Specialist Dentist *n* (%)
	13 (13.0)	40 (22.5)	13 (19.7)	3 (3.9)
Familiar with oral irrigator (Yes)	81 (81.0)	166 (92.7)	62 (93.9)	76 (100.0)
Knowledge of periodontal effects (Partial + Detailed)	67 (67.0)	164 (91.6)	66 (100.0)	73 (96.1)
Perceived effectiveness (Very effective + Effective)	66 (66.0)	134 (75.3)	57 (86.4)	64 (84.2)
Personal use (Regular + Occasional)	23 (23.0)	53 (29.6)	27 (40.9)	27 (35.5)
Recommends to patients (Yes)	68 (68.0)	144 (80.9)	61 (92.4)	62 (81.6)
Routine recommendation in practice (Yes)	69 (69.0)	132 (73.7)	47 (71.2)	49 (64.5)

Values represent number (percentage) within each professional group. Percentages were calculated within each group

Regarding undergraduate education, only 3.9% (*n* = 3) of specialist dentists reported receiving sufficient education about oral irrigators, which was the lowest rate among all groups, while 57.4% (*n* = 241) disagreed and 26.2% (*n* = 110) were uncertain (*p* < 0.001) (Table 2).

###  Awareness

No statistically significant difference was observed among professional groups regarding personal use (*p* = 0.107). A total of 30.9% of participants reported having used an oral irrigator at least once. Regular use was 9.0%, occasional use was 21.9%, and 69.1% reported never using an oral irrigator (Tables 1 and 2).

**Fig. 2:**
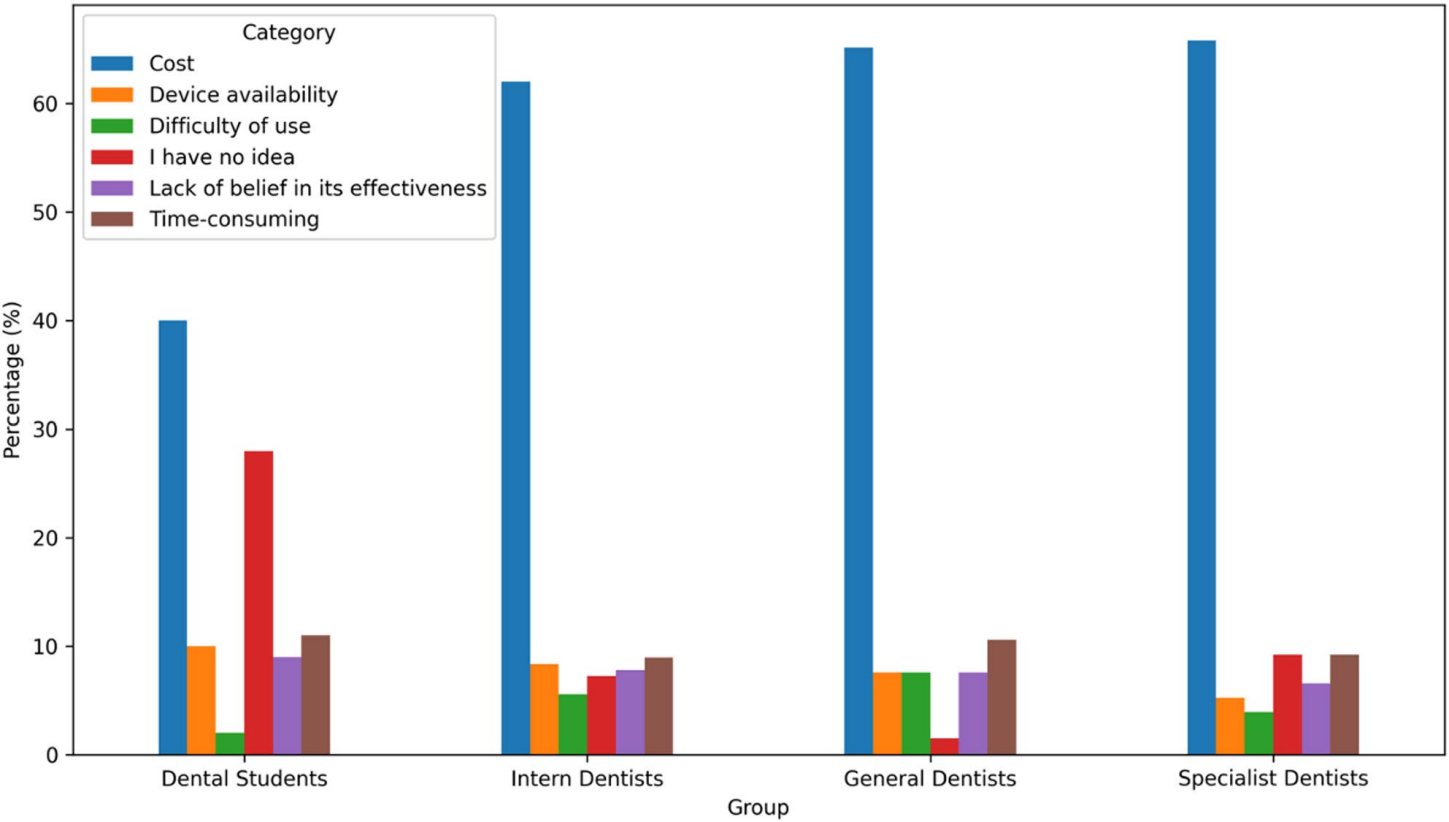
Barriers to oral irrigator use by group

The most frequently reported barrier to oral irrigator use was cost, cited by 58.0% (*n* = 244) of participants. Other reported barriers included time-consuming use (9.7%), device availability (8.1%), lack of belief in effectiveness (7.8%), and difficulty of use (4.8%). These differences were statistically significant between professional groups (*p* < 0.001). The most significant factor limiting the use of oral irrigators was cost, identified by 58.0% (*n* = 244) of the total study population, particularly among general dentists (65.2%) and specialist dentists (65.8%) (Fig. 2).

**Fig. 3:**
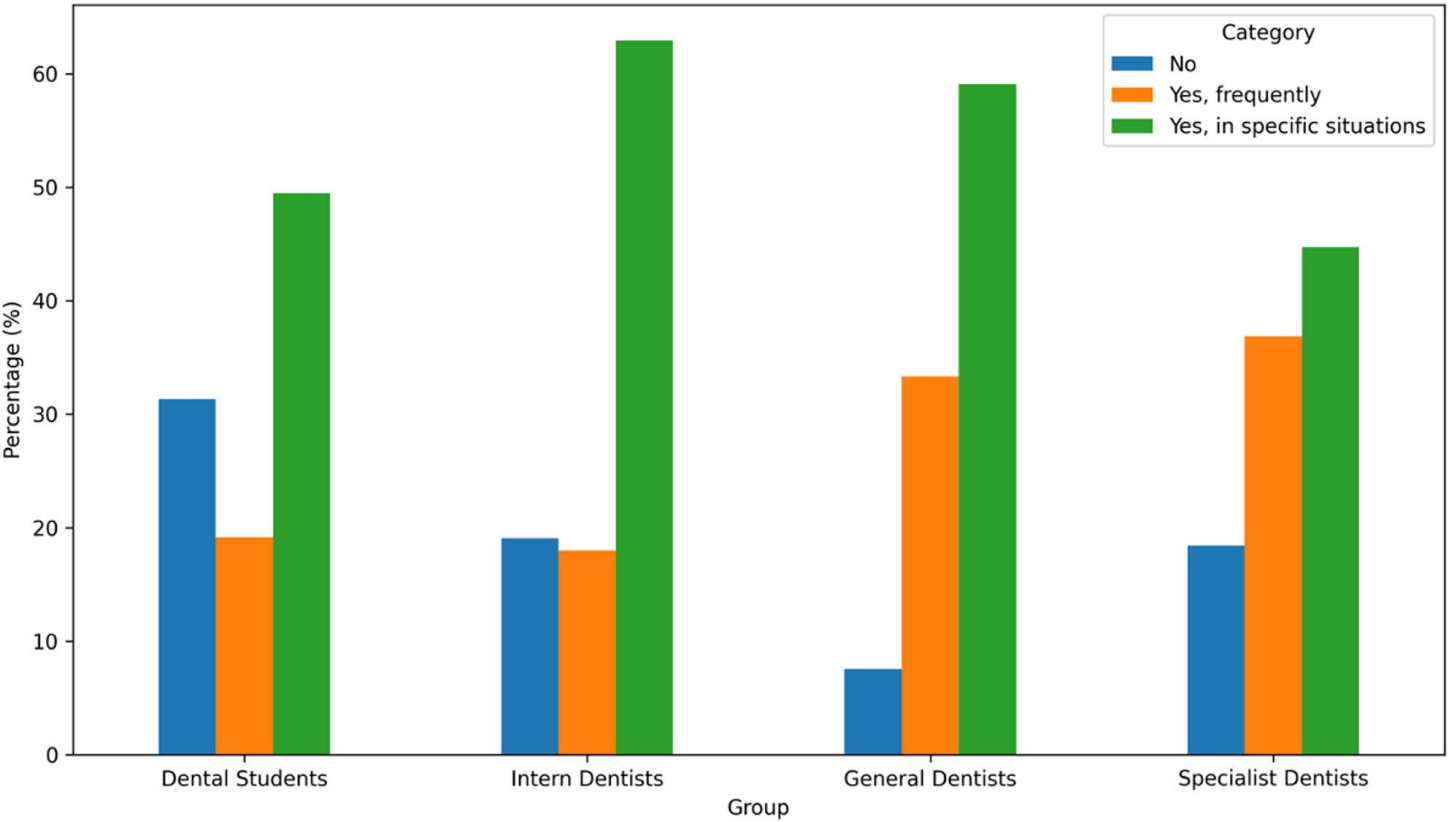
Frequency of recommending oral irrigator to patients by group

A significant difference was detected among professional groups regarding the frequency of recommending oral irrigators to patients (*p* < 0.001). The proportions of participants who reported recommending the device (either frequently or in specific situations) were 81.6% among specialist dentists, 92.4% among general dentists, 80.9% among intern dentists, and 68.7% among dental students (Fig. 3).

Participants identified several patient groups for whom oral irrigator use is especially beneficial. The most frequently cited groups were individuals with periodontal problems (69.8%), orthodontic patients (67.9%), and the general population (65.8%), followed by implant patients (57.7%), individuals with physical or intellectual disabilities (45.4%), and children (28.0%) (Table 3).

**Table 3:** Patient groups considered particularly beneficial for oral irrigator use

Patient group	*n*	%
Individuals with periodontal problems	294	69.8
Orthodontic patients	286	67.9
General population (everyone)	277	65.8
Implant patients	243	57.7
Individuals with physical and/or intellectual disabilities	191	45.4
Children	118	28.0
Other	26	6.2

### Attitude

A statistically significant difference was found regarding participants’ perception of the effectiveness of oral irrigator use (*p* = 0.016). Among participants, 18.3% considered it “very effective,” 58.1% “effective,” 17.9% “partially effective,” while 1.0% found it ineffective and 4.8% reported having no opinion (Table 2).

**Fig. 4:**
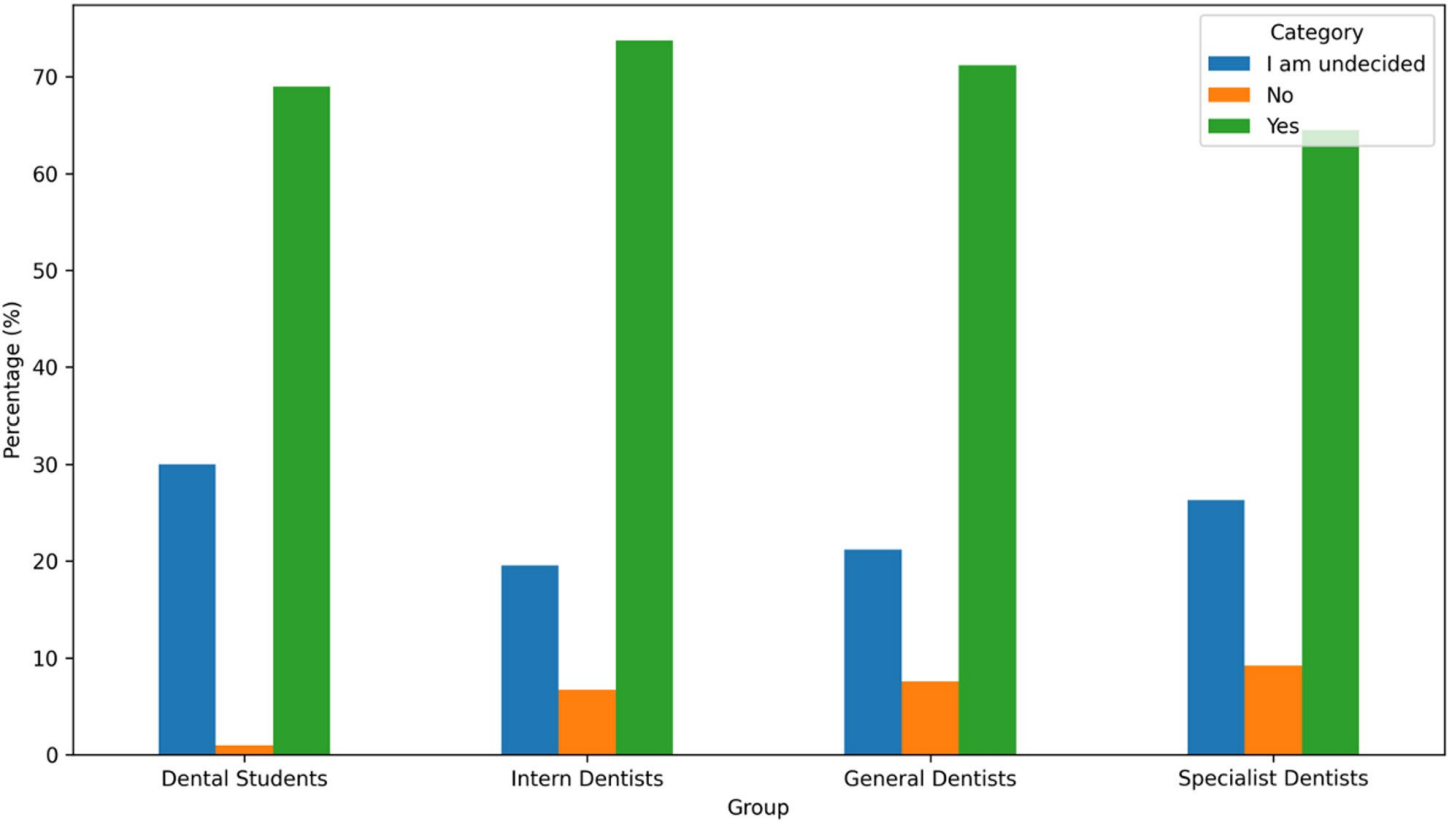
Recommendation of routine oral irrigator use by group

No statistically significant difference was found between professional groups regarding whether routine recommendation of oral irrigators should be included in clinical practice (*p* = 0.108). Overall, 70.5% of participants agreed, 5.9% disagreed, and 23.5% were undecided (Fig. 4).

## Discussion

Effective plaque control, particularly in interdental areas, is essential for maintaining periodontal health [26]. Although interdental brushes and traditional floss are commonly recommended, their routine use may be limited by patient-related factors, and oral irrigators have emerged as a practical adjunct for selected individuals [29–32]. 

In this context, the present study assessed familiarity, perceived effectiveness, and attitudes toward oral irrigator use across multiple stages of dental education and clinical practice. The findings demonstrated clear variability among groups, indicating that clinical experience plays a substantial role in shaping familiarity with and acceptance of adjunctive oral hygiene devices. While participants generally acknowledged the potential benefits of oral irrigators, inconsistencies emerged regarding personal use, understanding of mechanisms, and confidence in recommending them. Preclinical and early clinical students reported lower awareness and familiarity than practicing dentists, suggesting that patient care experience significantly contributes to professional confidence in integrating such devices into oral hygiene advice. Our findings indicate that awareness and use of oral irrigators remain inconsistent even among dental professionals and dental students. This pattern suggests that oral irrigators are still perceived as an ‘optional’ adjunct rather than a routine interdental cleaning aid, despite their potential benefits for specific patient groups. The observed variability across participant categories may reflect differences in clinical exposure, patient counselling responsibilities, and opportunities for hands-on training during professional development.

Statistical analyses further revealed significant group differences across several outcomes, underscoring the role of educational exposure in shaping oral hygiene decision-making. Although overall familiarity was high (82.9%), the strong group effect (*p* < 0.001) indicates that recognition alone does not translate into meaningful understanding. This pattern persisted in participants’ knowledge of periodontal benefits, while nearly all dentists reported partial or detailed understanding, this proportion was notably lower among students (67%), indicating a clear educational gradient. Significant differences in perceived effectiveness (*p* = 0.016) reinforce this gradient, with experienced clinicians showing greater confidence in oral irrigators’ utility. However, actual personal use remained low (12.4% regular use), highlighting a persistent gap between perceived value and behavioral adoption.

Notably, a common discrepancy in preventive behaviors is the gap between perceived effectiveness and regular personal use. In our sample, favorable perceptions did not consistently translate into routine adoption, which may be explained by practical barriers (e.g., cost, device accessibility, and perceived complexity), as well as uncertainty about indications and correct technique. This gap is clinically important because clinicians’ personal experience and confidence with a device may influence whether they recommend it to patients and how effectively they provide usage instructions.

Barriers such as cost, difficulty of use, and skepticism regarding effectiveness also varied significantly among groups (*p* < 0.001). Students most frequently cited limited access or awareness, further illustrating insufficient curricular exposure. Only 28.9% of participants reported receiving adequate education on oral irrigators, confirming a notable instructional deficit. Previous survey-based reports among dental professionals and students have similarly highlighted variability in the knowledge and routine use of oral hygiene adjuncts, indicating that awareness does not necessarily ensure consistent practice. Our findings extend this evidence by focusing specifically on oral irrigators and emphasizing the role of structured training and clear clinical indications in translating awareness into routine recommendation [27–28].

Consistent with previous studies assessing knowledge and practice regarding oral hygiene adjuncts, our results support the need for structured educational coverage of oral irrigators within dental curricula and continuing professional education. Integrating evidence-based indications (e.g., orthodontic appliances, implants, periodontal maintenance, and limited dexterity) with practical demonstrations may improve both competence and recommendation behaviors. Future studies should also evaluate whether targeted educational interventions increase correct knowledge, appropriate prescribing/recommendation patterns, and patient-level outcomes.

The study’s findings align with the narrative review and survey of Italian dental hygienists, which reported higher awareness and positive attitudes among experienced clinicians managing implant-supported prostheses [33]. Although the Italian study emphasized clinical performance around implants, our results similarly show that positive attitudes are not uniformly reflected among students, highlighting the importance of early educational reinforcement.

Comparable patterns have also been reported in community-based studies such as Sabbahi et al. [34], where younger and less experienced individuals demonstrated limited knowledge of interdental oral irrigators. Likewise, Yang et al. [35] demonstrated that structured oral hygiene training meaningfully improves attitudes and behaviors—an insight directly relevant to the educational gaps identified in our study.

Studies focusing on students’ awareness of advanced flossing techniques, such as the pilot work from Mangalore [36], further support our findings, reporting high conceptual familiarity but low usage rates. This mirrors the divide between theoretical knowledge and practical adoption observed among preclinical students in our cohort. Similarly, research among dentists in Bangalore [37] and the cross-sectional study by Mohamed et al. [28] underscore discrepancies between student and professional groups in familiarity and practice of interdental aids, reinforcing the notion that clinical experience is a key determinant of adoption.

The findings from Dharwad college students also demonstrate limited awareness of adjunctive cleaning devices among younger learners—a pattern aligning with our observation of reduced awareness and confidence among preclinical dental students [38]. Together, these studies collectively highlight the broad and persistent need for structured, evidence-based instruction on adjunctive oral hygiene methods at early stages of dental training.

This study has several limitations. First, participants were recruited using snowball sampling, which may introduce selection bias and limit the generalizability of the findings. Second, the results rely on self-reported responses, which are subject to recall and social desirability bias. Third, specialist dentists were analyzed as a single group because specific specialty fields were not recorded; therefore, potential differences in knowledge and recommendation patterns across specialties (e.g., periodontology) could not be examined. In addition, the online survey design may have constrained participation to individuals with greater digital access and interest in oral hygiene topics. Despite these limitations, the findings highlight an educational gap regarding oral irrigator indications and use among dental students and clinicians. Future studies should recruit representative samples, stratify respondents by specialty, and evaluate whether structured educational interventions and hands-on training improve knowledge, confidence in patient counselling, and appropriate recommendation behaviors, ideally linking these outcomes to patient-level oral health indicators. Additionally, the cross-sectional design does not allow causal inferences between education level and attitudes toward oral irrigator use.

Finally, our results showed significant group differences in identifying appropriate patient groups—especially orthodontic, implant, and periodontal patients—indicating that clinicians with greater experience are better equipped to personalize preventive strategies. The internal reliability of attitudinal items (Cronbach’s α = 0.64) further supports that participants’ perceptions reflect a coherent motivational construct.

## Conclusions

This study provides a detailed assessment of knowledge, awareness, and attitudes regarding oral irrigator use across different stages of dental education and clinical practice. The significant differences observed between students, general dentists, and specialists indicate that clinical exposure plays an important role in shaping familiarity, perceived effectiveness, and patient recommendation behaviors. Despite high general awareness, notable gaps were identified in detailed knowledge, personal use, and the adequacy of curriculum-based instruction, particularly among preclinical students.

These findings emphasize the need to enhance preventive dentistry training by integrating structured, evidence-based education on adjunctive oral hygiene devices. Strengthening educational exposure may support more confident and consistent clinical guidance, ultimately contributing to improved patient-centered oral hygiene practices.

## Supplementary Information

Below is the link to the electronic supplementary material.

Supplementary Material 1.

## Data Availability

The datasets generated and/or analysed during the current study are not publicly available due to institutional ethical restrictions but are available from the corresponding author on reasonable request.
