# Supplementary material for: Knowledge, awareness, and attitudes toward oral irrigator use among dentists and dental students: a cross-sectional study
Source: BMC Oral Health. 2026 Feb 26;26:578. doi: 10.1186/s12903-026-07956-w (PMC13041261; doi:10.1186/s12903-026-07956-w)
Supplement: Supplementary file 1 — Supplementary Material 1. [file 12903_2026_7956_MOESM1_ESM.pdf]

Questionnaire

- **Please select the option that best describes your current status:**
  a) *I am a dental student (third-year undergraduate student in the Faculty of Dentistry).*
  b) *I am a trainee dentist (fourth-year undergraduate student in the Faculty of Dentistry).*
  c) *I am a trainee dentist (fifth-year undergraduate student in the Faculty of Dentistry).*
  d) *I am a dentist.*
  e) *I am a specialist dentist.*
- **Do you have knowledge about oral irrigators (water flossers)?**
  a) *Yes*
  b) *No*
  c) *I am not sure*
- **How would you describe the primary function of an oral irrigator (water flosser)?**
  a) *An alternative to toothbrushing*
  b) *An adjunctive method that supports toothbrushing*
  c) *A device used only for orthodontic patients*
  d) *I have no idea*
- **Do you have knowledge regarding the effects of oral irrigators (water flossers) on periodontal health?**
  a) *Yes, I have detailed knowledge*
  b) *Yes, I have partial knowledge*
  c) *No*
- **In your opinion, for which patient groups are oral irrigators particularly beneficial?** *(You may select more than one option)*
  a) *Individuals undergoing orthodontic treatment*
  b) *Dental implant patients*
  c) *Individuals with periodontal problems*
  d) *Children*
  e) *Individuals with physical and/or intellectual disabilities*
  f) *General population (everyone)*
  g) *Other: …………………………*
- **To what extent do you consider oral irrigators to be an effective oral hygiene method?**
  a) *Very effective*
  b) *Effective*
  c) *Partially effective*
  d) *Ineffective*
  e) *I have no idea*
- **Do you think the routine use of oral irrigators should be recommended in dental practice?**
  a) *Yes*
  b) *No*
  c) *I am undecided*
- **Have you ever used an oral irrigator?**
  a) *Yes, I use it regularly.*
  b) *Yes, I use it occasionally.*
  c) *No, I have never used it.*
- **If you have used an oral irrigator, for what purpose did you use it?**
  a) *During orthodontic treatment*
  b) *For peri-implant care*
  c) *For controlling gingival bleeding/plaque*
  d) *For general oral hygiene*
  e) *I have not used it*
- **After using an oral irrigator, my mouth feels cleaner and fresher.**
  a) *I agree*
  b) *I disagree*
  c) *I am not sure*
  d) *I have not used it*
- **Oral irrigators are effective in reducing halitosis (bad breath).**
  a) *I agree*
  b) *I disagree*
  c) *I am not sure*
- **Do you recommend oral irrigators to your patients?**
  a) *Yes, frequently*
  b) *Yes, in specific situations*
  c) *No*
- **How do you think oral irrigator use affects patient compliance?**
  a) *Positively*
  b) *Neutral*
  c) *Negatively*
  d) *I have no idea*
- **What do you think is the most significant factor limiting the use of oral irrigators?**
  a) *Cost*
  b) *Device availability*
  c) *Difficulty of use*
  d) *Time-consuming*
  e) *Lack of belief in its effectiveness*
  f) *I have no idea*
- **Do you think you received sufficient education on oral irrigators during your undergraduate dental training?**
  a) *Yes*
  b) *No*
  c) *Partially*
- **I believe that the use of oral irrigators increases patients' motivation for maintaining oral hygiene.**
  a) *I agree*
  b) *I disagree*
  c) *I am not sure*
